# Synthesis of sulfonimidamides from sulfinamides by oxidation with *N*-chlorosuccinimide

**DOI:** 10.1186/1860-5397-3-25

**Published:** 2007-09-25

**Authors:** Olga García Mancheño, Carsten Bolm

**Affiliations:** 1 Institute of Organic Chemistry, RWTH Aachen University, Landoltweg 1, D-52056 Aachen, Germany

## Abstract

**Background:**

The synthesis of sulfonimidamides involves the nucleophilic substitution of a sulfonimidoyl chloride with an amine. However, only four chlorinating systems have been reported for the preparation of the sulfonimidoyl chloride intermediates. Whereas some of them have shown a rather limited substrate spectrum, the most versatile and commonly used *tert*-butyl hypochlorite is known to be explosive. To establish alternative methods for the synthesis of these molecules is therefore desirable.

**Results:**

The preparation of various *p*-tolylsulfonimidamides through the reaction of the corresponding *N*-protected *p*-tolylsulfinamides and a number of amines in the presence of *N*-chlorosuccinimide was achieved at room temperature in 50–97% yield.

**Conclusion:**

A convenient alternative procedure for the synthesis of sulfonimidamides from sulfinamides and various amines and sulfonamides using *N*-chlorosuccinimide as halogenating agent has been developed.

## Introduction

**Scheme 1:**
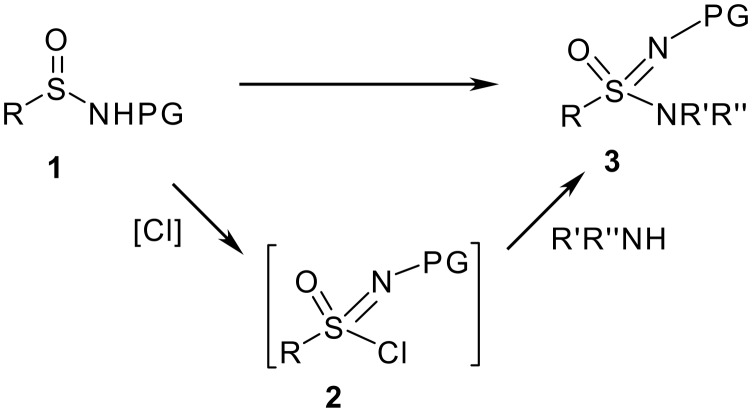
General synthesis of sulfonimidamides **3** from sulfinamides **1**.

Sulfonimidamides **3** are derivatives of sulfonic acid and analogous of sulfonamides, in which one oxygen has been replaced by a nitrogen group. They are known since 1962,[1] and a number of recent investigations focussed on both their reactivity and application in organic synthesis, such as nitrogen sources for metal-catalyzed nitrene transfer reactions, [2–5] and their biological activity, for instance as analogous of oncolytic sulfonylureas [6–8] or mimics of intermediates in protease and amidase reactions. [9] Only a few synthetic approaches for their preparation have been reported, the most direct and common route being the nucleophilic substitution of a sulfonimidoyl chloride **2** with an amine (Scheme 1).

Various chlorinating reagents can be applied for the synthesis of the respective sulfonimidoyl intermediates. Among them, and despite its explosive nature, *tert*-butyl hypochlorite is the most widely used one. [10–13] Other chlorinating agents present a rather limited substrate scope. For example, chlorine [14–17] is preferred for *N*-alkyl sulfinamides, reacting very violently with *N*-aryl derivatives. *N*-chlorobenzotriazole [15–16,18] is less efficient with bulky amines, and with chloramine-T or -N[3–4,19] only *N*-tosyl or -nosyl sulfonimidamides can be obtained. In addition, an alternative route to the intermediate *N*-tosyl or -nosyl sulfonimidoyl chlorides involves the reaction of sulfinyl chlorides with chloramine-T or -N.

In connection with our interest on the application of sulfonimidamides in organic synthesis,[20] we now aimed at exploring an alternative and general procedure for the synthesis of these molecules avoiding the use of potential explosive reagents.

## Results and discussion

**Scheme 2:**
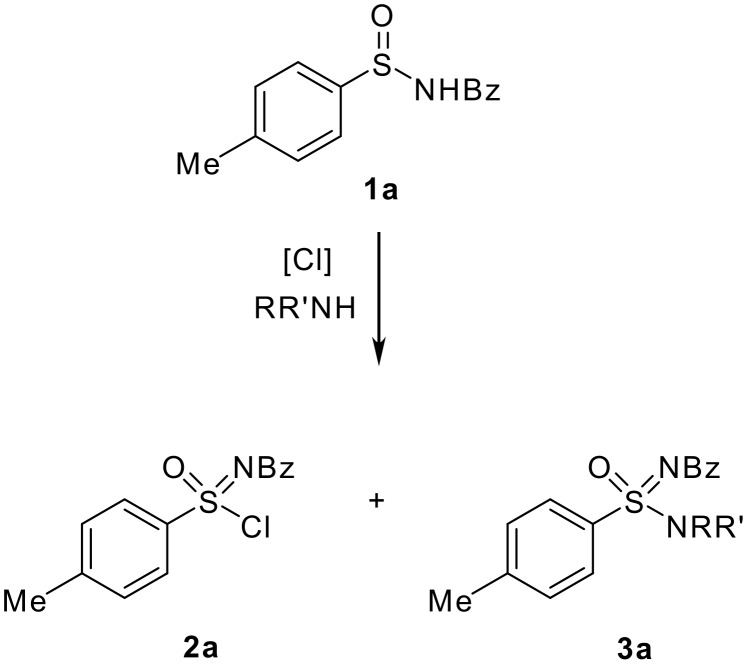
Synthesis of *N*-benzoyl sulfonimidamides **3a**.

For the preliminary screening, *N*-benzoyl sulfinamide **1a** was chosen as the model substrate (Scheme 2).

First, the reaction of **1a** with different halogenating agents was studied (Table 1).

**Table 1:** Halogenating agent effect on the synthesis of sulfonimidamides 3^a^

Entry	PG	SM	Halogenating agent	Nucleophile	Solvent	Yield of **2** (%)^b^	Yield of **3** (%)^b^

1	Bz	**1a**	Chloramine-T	--	MeCN	33	54
2	Bz	**1a**	Chloramine-T	--	MeCN/MS 4Å	30	53
3	Bz	**1a**	Chloramine-T	--	CH_2_Cl_2_	31	32
4	Bz	**1a**	Chloramine-T	--	Toluene	68	--
5	Bz	**1a**	Chloramine-T	TsNHNa	MeCN	--	91
6	Bz	**1a**	Chloramine-T	TsNHNa	THF	--	91
7	Bz	**1a**	*t*-BuOCl	TsNHNa	THF	20	73
8	Bz	**1a**	I_2_^c^	TsNHNa	MeCN	--	<10
9	Bz	**1a**	Bromamine-T	TsNHNa	MeCN	--	71
10	Bz	**1a**	NBS^d^	TsNHNa	MeCN	--	58
11	Bz	**1a**	NCS	TsNHNa	THF	--	85
12	Bz	**1a**	NCS	TsNHNa	MeCN	--	94
13	Bn	**1b**	NCS	TsNHNa	MeCN	--	56
14	Boc	**1c**	NCS	TsNHNa	MeCN	--	78

^a^ Reaction conditions: sulfinamide **1** (1.0 equiv), halogenating agent (1.2 equiv) and TsNHNa (2.0 equiv) in the desired dry solvent (0.1 M) at room temperature for 20 h. ^b^ Yield after column chromatography. ^c^ Use of 4 equiv of I_2_. ^d^ Okuma *et al*. mentioned the use of NBS and secondary amines for the preparation of sulfonimidamides from sulfinamides. However, no yield has ever been recorded. [21]

Starting point was the use of chloramine-T as the most common chlorinating reagent for such transformation. As hypothesized, the reaction involved the corresponding sulfonimidoyl chloride. Thus, in the reaction of **1a** with chloramine-T in acetonitrile both sulfonimidoyl chloride **2a** and sulfinamidamide **3a** were isolated in 33 and 54% yield, respectively (Table 1, Entry 1). The use of MS 4Å (1 g/mmol) did not improve this result, leading to a similar mixture of **2a** and **3a** after 20 h (Entry 2). Other solvents such as dichloromethane gave an unsatisfactory **2a**:**3a** ratio of 1:1 (31 and 32% yield, respectively; Entry 3). Moreover, the reaction in toluene gave exclusively **2a** in 68% yield after 24 h (Entry 4). Gratifyingly, using a combination of chloramine-T and TsNHNa, the desired sulfonimidamide **3a** was obtained selectively and in high yield (91%) in both acetonitrile and THF as solvents (Table 1, Entries 5 and 6). The reaction with the highly reactive *t-*BuOCl was surprisingly less efficient, leading to **3a** in 73% yield after 20 h, along with unreacted sulfonimidoyl chloride **2a** (20%, Table 1, Entry 7). Other halogenating agents, such as I_2_, bromamine-T, or NBS,[21] were tested as well (Table 1, Entries 8–10), but they exhibited a significantly lower efficiency than the previous chlorinating agents.

Since *N*-chlorosuccinimide (NCS) had been applied for the oxidation of 4-(methylthio)morpholine towards the synthesis of diazasulfonium salts[22] and the preparation of dialkylamino succinimidosulfonium salts from sulfenamides,[23] this non-explosive and easy to handle oxidizing agent was tested next. To our delight, **1a** and NCS reacted well, and in combination with TsNHNa in acetonitrile sulfonimidamide **3a** was obtained in excellent yield (94%, Table 1, Entry 12). Noteworthy, the qualitative formation of sulfonimidoyl chloride **2a** and its conversion to sulfonimidamide **3a** could easily be followed by TLC.

Subsequently, the role of the substituent at the sulfinamide nitrogen was examined. The reactivity of *N*-benzoyl, -benzyl and -*tert*-butyl carbamate protected sulfinamides **1a-c**, which were prepared according to literature procedures from NH_2_-free *p*-tolylsulfinamide using *n*-BuLi and the corresponding anhydride[24] or by reaction of *p*-tolylsulfinyl chloride with BnNH_2_, was compared in the reaction with NCS and TsNHNa in CH_3_CN at room temperature (Table 1, Entries 12–14).

The best result was obtained with *N*-benzoyl sulfinamide **1a** (94%, Table 1, Entry 12). However, reaction of *N*-Boc derivative **1c** also gave the desired product **3c** in good yield (78%, Entry 14). On the other hand, *N*-benzyl derivative **1b** led to **3b** in only moderate 56% yield (Entry 13). Therefore, benzoyl was regarded as the *N*-protecting group of choice for the following studies.

In order to establish the generality of this method, the reaction of **1a** with different amines and amides was next investigated (Table 2).

**Table 2:** Amine scope^a^

Entry	RR'NH/Base	Product	R/R'	Yield of **3** (%)^b^

1	NsNHNa	**3d**	Ns/H	86
2	ThphNHNa	**3e**	Thph/H	94
3	BusNHNa	**3f**	Bus/H	50 (28)^c^
4	H_2_NCN/*t*-BuOK	**3g**	CN/H	85
5	PhNH_2_	**3h**	Ph/H	94
6	Me_2_NH	**3i**	Me/Me	97
7	(TMS)_2_NH	**3j**	H/H	89

^a^ Reaction conditions: sulfinamide **1a** (1.0 equiv), NCS (1.2 equiv) and RR'NH/Base (2.0 equiv) in dry acetonitrile (0.1 M) at room temperature. ^b^ Yield after column chromatography. ^c^ Yield of **2a** after column chromatography in brackets.

*p*-Nitrobenzenesulfonyl and thiophenesulfonylamide sodium salts (NsNHNa and ThphNHNa) were reacted with **1a** in the presence of NCS to yield sulfonimidamides **3** in good yields (86 and 94%, Table 2, Entries 1 and 2, respectively). In contrast, when the bulky *tert*-butylsulfonylamide sodium salt (BusNHNa) was used (Entry 3), the reaction was less efficient. In that case, the desired product **3f** was isolated in only moderate yield (50%), together with unreacted sulfonimidoyl chloride **2a** (28%), even after prolonged reaction times (24 h). The weakly basic cyanogen amine (pKa ~ 17), which had previously been used in the formation of *N*-cyano sulfilimines from sulfides using NBS as halogenating agent,[25] was also able to undergo the reaction in the presence of *t-*BuOK (85%, Table 2, Entry 4). Finally, the more reactive aniline, dimethylamine and hexamethyldisilazane (HMDS) were successfully employed. Even in the absence of an additional base the corresponding products were obtained after short reaction time (2–4 h) in 89–97% yield (Table 2, Entries 5–7).

**Scheme 3:**
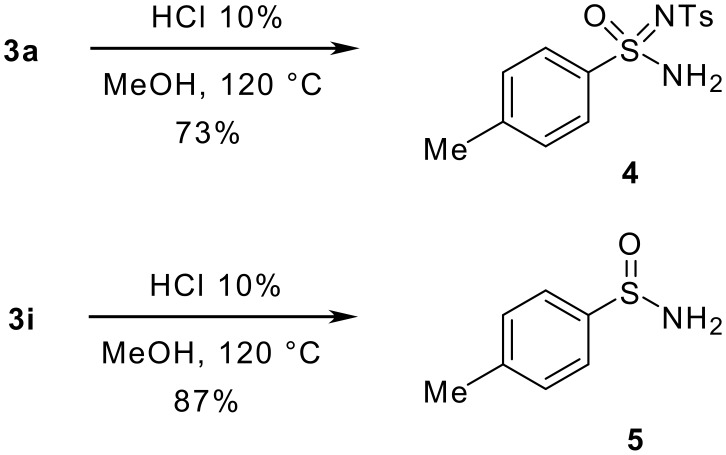
Cleavage of the *N*-benzoyl group.

Ultimately, the cleavage of the *N*-benzoyl group in **3a** was performed (Scheme 3). As expected, the exclusive formation of the most stable regioisomer **4** was observed (73% yield). On the other hand, under the same reaction conditions the attempted deprotection of **3i** gave sulfinamide **5** in good yield (87%) as a result of both *N*-benzoyl cleavage and elimination of the protonated dimethyl amino group.

In conclusion, we have described a convenient procedure for the synthesis of sulfonimidamides from sulfinamides using a variety of amines and *N*-chlorosuccinimide as oxidant. The reaction involves sulfonimidoyl chlorides formed in situ, which can be isolated depending on the reaction conditions. The cleavage of the *N*-benzoyl group has been achieved in the case of *N*-tosyl derivative **3a**. In contrast, the selective deprotection of *N*-benzoyl sulfonimidamides derived from secondary amine **3i** remained unsuccessful due to the concomitant elimination of the substituted amine group under the normal acidic conditions used.

## Experimental

[See Supporting Information File 1]

## Supporting Information

File 1 Synthesis of Sulfonimidamides from Sulfinamides by Oxidation with *N*-Chlorosuccinimide. Experimental Section. Experimental procedures, characterization of new compounds and ^1^H and ^13^C NMR spectra.
