# Supplementary material for: Synthesis of sulfonimidamides from sulfinamides by oxidation with N-chlorosuccinimide
Source: Beilstein J Org Chem. 2007 Sep 25;3:25. doi: 10.1186/1860-5397-3-25 (PMC2151070; doi:10.1186/1860-5397-3-25)

SUPPORTING INFORMATION FOR
Synthesis of Sulfonimidamides from Sulfinamides by Oxidation with *N*-Chlorosuccinimide

Olga García Mancheño and Carsten Bolm*

Institute of Organic Chemistry, RWTH Aachen University, Landoltweg 1, D-52056 Aachen, Germany

e-mail: Carsten.Bolm@oc.rwth-aachen.de

Table of Contents

- General S1
- General procedure for the synthesis of sulfonimidamides S1
- Analytical data for compounds **2a** and **3a-j** S2
- General procedure for the cleavage of the *N*-benzoyl group S5
- Analytical data for compounds **4** and **5** S5
- References S6
- ^{1}H and ^{13}C spectra S7

General: ^{1}H and ^{13}C NMR spectra were recorded in $CDCl_3$, CD_3OD or acetone-d_6 on a Varian Inova 400 or a Varian Mercury 300 spectrometer (400 and 100 MHz, and 300 and 75 MHz, respectively). Chemical shifts are given in ppm and spin-spin coupling constants, *J*, are given in Hz. Melting points were determined in open-end capillary tubes on a Büchi B-540 melting point apparatus and are uncorrected. Microanalyses were obtained with a Vario EL element analyzer. Mass spectra were acquired on a Varian MAT 212 spectrometer. IR spectra were taken on a Perkin-Elmer FT/IR 1760 and were recorded as KBr pellets or in solution. All reagents were purchased from commercial suppliers and used without further purification. Amide sodium salts were obtained upon treatment of the corresponding sulfonamide with NaH (1 equiv, 60% in mineral oil). Sulfinamides **1a-c** were prepared according to literature procedures from NH_2-free *p*-tolylsulfinamide **5** using *n*-BuLi and the corresponding anhydride,[1] or **1b** by reaction of *p*-tolylsulfinyl chloride with $BnNH_2$.

General procedure for the synthesis of sulfonimidamides 3:

To a stirring solution of sulfinamide **1** (1.0 mmol) and amine/amino salt (2.0-5.0 mmol) or H_2NCN (88.2 mg, 2.1 mmol) and *t*-BuOK (235.6 mg, 2.0 mmol) in dry acetonitrile

under argon at room temperature, NCS (160.3 mg, 1.2 mmol) was added. Once the starting material was consumed (monitored by TLC), the reaction mixture was concentrated under reduced pressure and the residue was purified by flash column chromatography.

***N*-(Benzoyl)-*p*-toluenesulfonimidoyl chloride (2a):**[2]

Pale yellow solid. Mp. 102-104 ºC (Lit.[2] mp. 105-106 ºC); ^{1}H NMR (300 MHz, $CDCl_3$): δ 8.18-8.10 (m, 4H), 7.58 (tt, *J* = 7.4, 1.4 Hz, 1H), 7.50-7.42 (m, 4H), 2.53 (s, 3H); ^{13}C NMR (75 MHz, $CDCl_3$): δ 170.9 (C=O), 146.9 (C), 140.2 (C), 134.2 (C), 133.3 (CH), 130.4 (2 x CH), 129.8 (2 x CH), 128.4 (2 x CH), 127.2 (2 x CH), 21.9 (CH_3); MS (EI+), *m/z* (relative intensity): 294 [M^+, 18], 258 [M^+-Cl, 5], 190 [$(M+H)^+$-Bz, 28], 155 [40], 105 [100].

***N*-(Benzoyl)-*N'*-(*p*-methylbenzenenesulfonyl)-*p*-toluenesulfonimidamide (3a):**

Following the general procedure, the reaction of sulfinamide **1a** with NCS and TsNHNa gave **3a** as a white solid (94%). Chromatography: gradient of ethyl acetate/pentane 1:4 to 1:1. Mp. >200 ºC (decomp.); ^{1}H NMR (400 MHz, acetone-d_6): δ 7.84 (d, *J* = 7.4 Hz, 2H), 7.66 (d, *J* = 8.5 Hz, 2H), 7.58 (d, *J* = 8.5 Hz, 2H), 7.31 (t, *J* = 7.4 Hz, 1H), 7.16 (t, *J* = 7.4 Hz, 2H), 7.05 (d, *J* = 8.5 Hz, 2H), 6.89 (d, *J* = 8.5 Hz, 2H), 2.20 (s, 3H), 2.11 (s, 3H); ^{13}C NMR (100 MHz, acetone-d_6): δ 173.1 (C=O), 142.6 (C), 141.5 (C), 141.0 (C), 139.8 (C), 136.7 (C), 131.5 (CH), 129.2 (2 x CH), 128.9 (2 x CH), 128.6 (2 x CH), 127.5 (2 x CH), 126.8 (2 x CH), 126.4 (2 x CH), 20.6 (CH_3), 20.5 (CH_3); IR (KBr): ν 3257, 1596, 1419, 1311, 1263, 1152, 1105, 1079, 1024 cm^{-1}; MS (ESI-), *m/z*: 427 [$(M-H)^-$]; Calcd. for $C_{21}H_{20}N_2O_4S_2·H_2O$: C, 56.48; H, 4.97 N, 6.27; found C, 56.31; H, 4.83; N, 5.90.

***N*-(Benzyl)-*N'*-(*p*-methylbenzenenesulfonyl)-*p*-toluenesulfonimidamide (3b):**

Following the general procedure, the reaction of sulfinamide **1b** with NCS and TsNHNa gave **3b** as a white solid (50%). Chromatography: gradient of ethyl acetate/pentane 1:4 to 1:1. Mp. 135-137 ºC; ^{1}H NMR (400 MHz, acetone-d_6): δ 7.78 (d, *J* = 8.2 Hz, 2H), 7.73 (d, *J* = 8.2 Hz, 2H), 7.39 (d, *J* = 8.2 Hz, 2H), 7.32-7.24 (m, 6H), 4.18 (AB system, *J* = 14.5 Hz, 1H), 4.12 (AB system, *J* = 14.5 Hz, 1H), 2.43 (s, 3H), 2.39 (s, 3H); ^{13}C NMR (100 MHz, acetone-d_6): δ 144.2 (C), 142.2 (C), 141.7 (C), 136.6 (C), 136.3 (C), 129.6 (2 x CH), 128.9 (2 x CH), 128.3 (CH), 128.0 (2 x CH), 127.5 (4 x CH), 126.5 (2 x CH), 46.1 (CH_2), 20.7 (CH_3), 20.6 (CH_3); IR (KBr): ν 3256, 1596, 1419, 1311, 1263, 1152, 1105, 1079 cm^{-1}; MS (EI+), *m/z* (relative intensity): 415 [$(M+H)^+$, 1], 309 [M^+-

NBn, 1], 138 [27], 106 [100]; Calcd. for $C_{21}H_{22}N_2O_3S_2$: C, 60.84; H, 5.35 N, 6.76; found C, 60.89; H, 5.28; N, 6.73.

***N*-[*tert*-Butoxycarbonyl)amino]-*N'*-(*p*-methylbenzenenesulfonyl)-*p*-toluenesulfon-imidamide (3c):**

Following the general procedure, the reaction of sulfinamide **1c** with NCS and TsNHNa gave **3c** as a white solid (78%). Chromatography: gradient of ethyl acetate/pentane 1:1 to ethyl acetate. Mp. >147 ºC (decomp.); ^{1}H NMR (300 MHz, CD_3OD): δ 7.57 (d, *J* = 8.2 Hz, 2H), 7.53 (d, *J* = 8.2 Hz, 2H), 7.11 (d, *J* = 7.8 Hz, 2H), 7.08 (d, *J* = 7.8 Hz, 2H), 2.34 (s, 3H), 2.33 (s, 3H), 1.23 (s, 9H); ^{13}C NMR (75 MHz, CD_3OD): δ 159.0 (C=O), 142.2 (C), 141.8 (C), 140.5 (C), 139.1 (C), 128.4 (2 x CH), 128.3 (2 x CH), 127.1 (2 x CH), 126.4 (2 x CH), 79.7 (C), 27.0 (3 x CH_3), 20.0 (CH_3); IR (KBr): ν 2979, 1646, 1299, 1152, 1091, 1050 cm^{-1}; MS (ESI-), *m/z*: 423 [(M-H)$^-$]; Calcd. for $C_{19}H_{24}N_2O_5S_2$·5/3H_2O: C, 50.20; H, 6.06 N, 6.16; found C, 50.13; H, 5.71 N, 5.76.

***N*-(Benzoyl)-*N'*-(*p*-nitrobenzenesulfonyl)-*p*-toluenesulfonimidamide (3d):**

Following the general procedure, the reaction of sulfinamide **1a** with NCS and NsNHNa gave **3d** as a white solid (86%). Chromatography: gradient of dichloromethane/acetone 4:1 to 1:1. Mp. >240 ºC (decomp.); ^{1}H NMR (400 MHz, acetone-d_6): δ 7.90 (s, 4H), 7.73 (d, *J* = 8.5 Hz, 2H), 7.71 (d, *J* = 8.5 Hz, 2H), 7.28 (t, *J* = 8.5 Hz, 2H), 7.16-7.06 (m, 4H), 2.21 (s, 3H); ^{13}C NMR (100 MHz, acetone-d_6): δ 172.8 (C=O), 150.1 (C), 148.8 (C), 142.5 (C), 140.2 (C), 136.7 (C), 131.3 (CH), 129.0 (2 x CH), 128.8 (2 x CH), 128.2 (2 x CH), 127.4 (2 x CH), 126.5 (2 x CH), 123.3 (2 x CH), 20.6 (CH_3); IR (KBr): ν 3103, 2924, 1605, 1531, 1325, 1152, 1048 cm^{-1}; MS (ESI-), *m/z*: 458 [(M-H)$^-$]; Calcd. for $C_{20}H_{17}N_3O_6S_2$·3/2 H_2O: C, 49.37; H, 4.14 N, 8.64; found C, 49.56; H, 4.05; N, 8.37.

***N*-(Benzoyl)-*N'*-(2-thiophenesulfonyl)-*p*-toluenesulfonimidamide (3e):**

Following the general procedure, the reaction of sulfinamide **1a** with NCS and ThphSO_2NHNa gave **3e** as a white solid (94%). Chromatography: gradient of dichloromethane/acetone 4:1 to 1:1. Mp. >230 ºC (decomp.); ^{1}H NMR (300 MHz, acetone-d_6): δ 7.98 (d, *J* = 8.4 Hz, 2H), 7.78 (d, *J* = 8.4 Hz, 2H), 7.49 (dd, *J* = 5.0, 1.5 Hz, 1H), 7.45-7.39 (m, 2H), 7.34-28 (br t, *J* = 7.4 Hz, 2H), 7.18 (d, *J* = 8.2 Hz, 2H), 6.83 (dd, *J* = 5.0, 3.7 Hz, 1H), 2.34 (s, 3H); ^{13}C NMR (75 MHz, acetone-d_6): δ 173.5 (C=O), 145.8 (C), 142.8 (C), 139.8 (C), 136.8 (C), 131.6 (CH), 130.7 (CH), 130.1 (CH), 129.3 (2 x CH), 129.0 (2 x CH), 127.7 (2 x CH), 126.4 (2 x CH), 126.2 (CH), 20.5 (CH_3); IR (KBr): ν 1606, 1571, 1327, 1286, 1050 cm^{-1}; MS (ESI-), *m/z*: 419 [M$^-$];

Calcd. for $C_{18}H_{16}N_2O_4S_3·4/3\ H_2O$: C, 48.63; H, 4.23 N, 6.30; found C, 48.86; H, 4.25; N, 6.01.

***N*-(Benzoyl)-*N'*-(*tert*-butylsulfonyl)-*p*-toluenesulfonimidamide (3f):**

Following the general procedure, the reaction of sulfinamide **1a** with NCS and BusNHNa gave **3f** as a white solid (50%) and sulfonimidoyl chloride **2a** (28%). Chromatography: gradient of dichloromethane/acetone 4:1 to 1:1. Mp. >200 ºC (decomp.); ^{1}H NMR (400 MHz, acetone-d$_6$): δ 8.11 (d, *J* = 8.2 Hz, 2H), 8.02 (d, *J* = 8.2 Hz, 2H), 7.45 (t, *J* = 7.1 Hz, 1H), 7.38-7.30 (m, 4H), 2.40 (s, 3H), 1.31 (s, 9H); ^{13}C NMR (100 MHz, acetone-d$_6$): δ 173.0 (C=O), 142.7 (C), 141.0 (C), 136.9 (C), 131.5 (CH), 129.2 (2 x CH), 129.1 (2 x CH), 127.7 (2 x CH), 126.1 (2 x CH), 58.9 (C), 23.8 (3 x CH_3), 20.1 (CH_3); IR (KBr): ν 2929, 1598, 1556, 1346, 1100 cm^{-1}; MS (ESI-), *m/z*: 393 [(M-H)$^-$]; Calcd. for $C_{18}H_{22}N_2O_4S_2·5/3H_2O$: C, 50.92; H, 6.01; N, 6.60; found C, 50.53; H, 5.69; N, 6.26.

***N*-(Benzoyl)-*N'*-(cyano)-*p*-toluenesulfonimidamide (3g):**

Following the general procedure, the reaction of sulfinamide **1a** with NCS, H_2NCN and *t*-BuOK gave **3g** as a white solid (85%). Chromatography: gradient of dichloromethane/acetone 4:1 to 1:1. Mp. >126 ºC (decomp.); ^{1}H NMR (300 MHz, acetone-d$_6$): δ 8.11 (d, *J* = 8.2 Hz, 2H), 7.92 (d, *J* = 8.2 Hz, 2H), 7.45 (t, *J* = 7.3 Hz, 1H), 7.39-7.28 (m, 4H), 2.37 (s, 3H); ^{13}C NMR (75 MHz, acetone-d$_6$): δ 173.1 (C=O), 142.2 (C), 140.8 (C), 137.5 (C), 131.1 (CH), 129.1 (2 x CH), 129.0 (2 x CH), 127.6 (2 x CH), 126.6 (2 x CH), 116.0 (CN), 20.5 (CH_3); IR (KBr): ν 2183, 1603, 1567, 1328 cm^{-1}; MS (ESI-), *m/z*: 298 [(M-H)$^-$]; Calcd. for $C_{15}H_{13}N_3O_2S·5/3H_2O$: C, 54.70; H, 5.00 N, 12.76; found C, 54.31; H, 5.18; N, 13.02.

***N*-(Benzoyl)-*N'*-(phenyl)-*p*-toluenesulfonimidamide (3h):**[3]

Following the general procedure, the reaction of sulfinamide **1a** with NCS and aniline gave **3h** as a white solid (94%). Chromatography: gradient of ethyl acetate/pentane 1:10 to 1:4. Mp. 169-172 ºC (Lit.[3] mp. 173-174 ºC); ^{1}H NMR (400 MHz, acetone-d$_6$): δ 9.69 (br s, 1H, NH), 8.08 (d, *J* = 8.5 Hz, 2H), 7.87 (d, *J* = 8.5 Hz, 2H), 7.52 (t, *J* = 7.4 Hz, 1H), 7.41 (t, *J* = 7.6 Hz, 2H), 7.34 (d, *J* = 8.5 Hz, 2H), 7.27-7.19 (m, 4H), 7.10-7.04 (m, 1H), 2.35 (s, 3H); ^{13}C NMR (100 MHz, acetone-d$_6$): δ 171.4 (C=O), 144.1 (C), 136.6 (C), 136.1 (C), 131.9 (CH), 129.7 (2 x CH), 129.2 (2 x CH), 129.0 (2 x CH), 127.9 (2 x CH), 127.8 (2 x CH), 124.9 (CH), 121.5 (2 x CH), 20.6 (CH_3); IR (KBr): ν 3420, 1602, 1329, 1286, 954 cm^{-1}; MS (EI+), *m/z* (relative intensity): 350 [M$^+$, 63], 258

[M^+-NHPh, 81], 105 [100]; Calcd. for $C_{20}H_{18}N_2O_2S$: C, 68.55; H, 5.18 N, 7.99; found C, 68.60; H, 5.46; N, 8.11.

***N*-(Benzoyl)-*N'*,*N'*-(dimethyl)-*p*-toluenesulfonimidamide (3i):**

Following the general procedure, the reaction of sulfinamide **1a** with NCS and dimethylamine gave **3i** as a white solid (97%). Chromatography: gradient of ethyl acetate/pentane 1:4 to 1:1. Mp. 95-96 ºC; ^{1}H NMR (300 MHz, acetone-d$_6$): δ 8.15 (d, *J* = 8.2 Hz, 2H), 7.86 (d, *J* = 8.2 Hz, 2H), 7.55 (t, *J* = 7.3 Hz, 1H), 7.50-7.41 (m, 4H), 2.84 (s, 6H), 2.45 (s, 3H); ^{13}C NMR (75 MHz, acetone-d$_6$): δ 171.4 (C=O), 144.0 (C), 136.5 (C), 133.0 (C), 131.8 (CH), 129.8 (2 x CH), 129.1 (2 x CH), 128.0 (2 x CH), 127.9 (2 x CH), 36.8 (2 x CH_3), 20.6 (CH_3); IR (KBr): ν 3015, 1636, 1454, 1283, 1142, 953 cm^{-1}; MS (EI+), *m/z* (relative intensity): 303 [M^+, 8], 259 [M^+-NMe_2, 40], 105 [100]; Calcd. for $C_{16}H_{18}N_2O_2S$: C, 63.55; H, 6.00 N, 9.26; found C, 63.66; H, 5.79; N, 9.24.

***N*-(Benzoyl)-*p*-toluenesulfonimidamide (3j):**[3]

Following the general procedure, the reaction of sulfinamide **1a** with NCS and hexamethyldisilazane gave **3j** as a white solid (89%). Chromatography: gradient of ethyl acetate/pentane 1:4 to 1:1. Mp. 137-138 ºC (Lit.[3] mp. 139-141 ºC); ^{1}H NMR (400 MHz, acetone-d$_6$): δ 8.05 (d, *J* = 8.5 Hz, 2H), 7.91 (d, *J* = 8.5 Hz, 2H), 7.49 (t, *J* = 7.3 Hz, 1H), 7.42-7.35 (m, 4H), 7.20 (br s, 2H), 2.40 (s, 3H); ^{13}C NMR (100 MHz, acetone-d$_6$): δ 171.7 (C=O), 143.4 (C), 139.8 (C), 136.4 (C), 131.6 (CH), 129.4 (2 x CH), 128.9 (2 x CH), 127.8 (2 x CH), 126.8 (2 x CH), 20.6 (CH_3); MS (EI+), *m/z* (relative intensity): 275 [$(M+H)^+$, 7], 197 [7], 108 [100].

General procedure for the cleavage of the *N*-benzoyl group:

To a solution of **3** (0.15 mmol) in MeOH (0.6 mL), a 10% HCl aq. solution (0.6 mL) was added at room temperature. The mixture was heated at 120 ºC in a sealed tube for 16 h. Saturated aqueous Na_2HCO_3 was added, extracted with CH_2Cl_2 (3 x 1 mL), dried over $MgSO_4$ and concentrated to dryness. The residue was purified by flash chromatography

***N*-(*p*-Toluenesulfonyl)-*p*-toluenesulfonimidamide (4):**[4]

Following the general procedure, the reaction of sulfonimidamide **3a** with 10% HCl solution in MeOH gave **4** as a white solid (73%). Chromatography: ethyl acetate/pentane 1:1. Mp. 164-165 ºC (Lit.[4] mp. 164-164.5 ºC); ^{1}H NMR (400 MHz,

acetone-d_6): δ 7.64 (d, J = 8.2 Hz, 2H), 7.52 (d, J = 8.2 Hz, 2H), 7.22 (d, J = 8.0 Hz, 2H), 7.12 (d, J = 8.0 Hz, 2H), 6.92 (br s, 2H, NH_2), 2.29 (s, 3H), 2.25 (s, 3H); ^{13}C NMR (100 MHz, acetone-d_6): δ 143.8 (C), 142.0 (C), 141.7 (C), 139.3 (C), 129.4 (2 x CH), 128.8 (2 x CH), 126.8 (2 x CH), 126.4 (2 x CH), 20.6 (CH_3), 20.5 (CH_3); MS (EI+), *m/z* (relative intensity): 325 [$(M+H)^+$, 7], 308 [M^+-NH_2, 4], 261 [100], 154 [91] 108 [86].

***p*-Tolylsulfinamide (5):**[5,6]

Following the general procedure, the reaction of sulfonimidamide **3i** with 10% HCl solution in MeOH gave **5** as a white solid (87%). Chromatography: ethyl acetate/pentane 1:1. Mp. 113-114 ºC (Lit.[5] mp. 113 ºC); ^1H NMR (300 MHz, acetone-d_6): δ 7.78 (d, J = 8.4 Hz, 2H), 7.36 (d, J = 8.4 Hz, 2H), 6.47 (br s, 2H, NH_2), 2.41 (s, 3H); ^{13}C NMR (75 MHz, acetone-d_6): δ 142.4 (C), 141.6 (C), 129.3 (2 x CH), 126.0 (2 x CH), 20.4 (CH_3).

References

1. García-Ruano J, Alonso R, Zarzuelo MM, Noheda P: *Tetrahedron: Asymmetry.* 1995, **6:**1133-1142.

2. Levchenko ES, Berzina IN, Kirsanov AV: *Zh. Org. Khim.* 1965, **1:**1251-1255.

3. Leca D, Toussaint A, Mareau C, Fensterbank L, Lacôte E, Malacria M: *Org. Lett.* 2004, **6:**3573-3575.

4. Di Chenna PH, Robert-Peillard F, Dauban P, Dodd RH: *Org. Lett.* 2004, **6:**4503-4505.

5. García Ruano JL, Alemán J, Fajardo C, Parra A: *Org. Lett.* 2005, **7:**5493-5496.

6. Davis FA, Zhang Y, Andemichae Y, Fang T, Fanelli DL, Zhang H: *J. Org. Chem.* 1999, **64:**1403-1406.

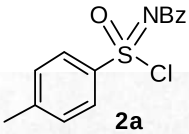

2a

300 MHz, $CDCl_3$

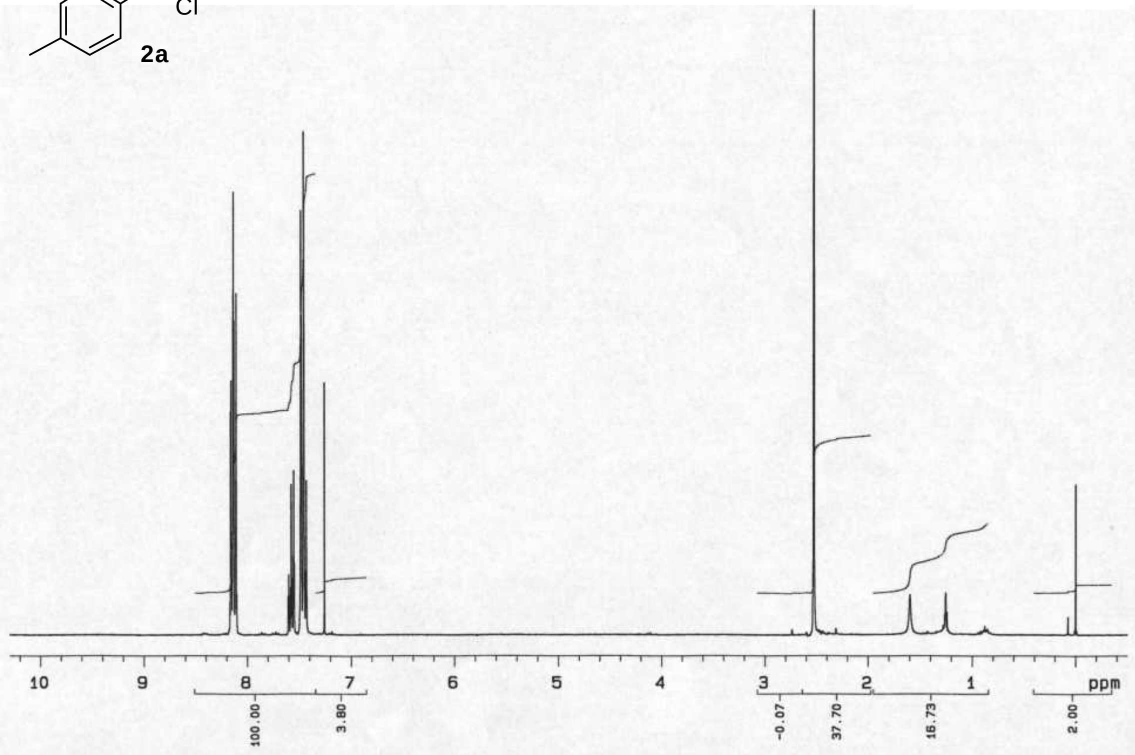

75 MHz, $CDCl_3$

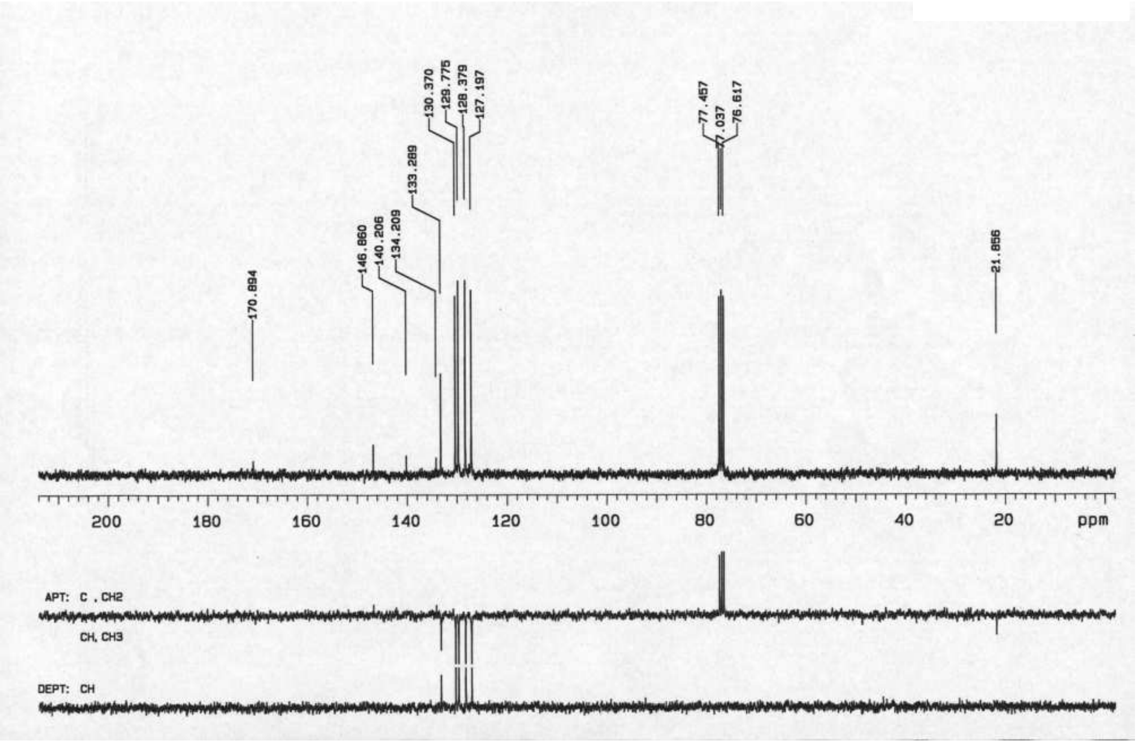

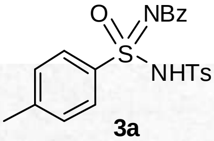

3a

400 MHz, acetone-d_6

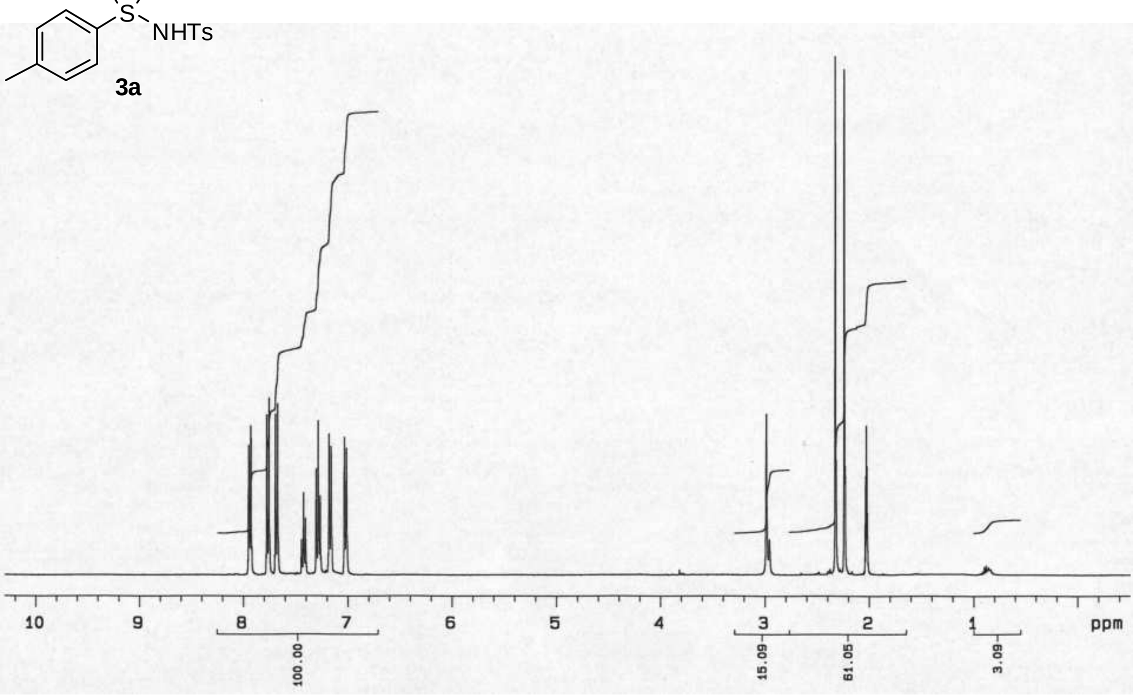

100 MHz, acetone-d_6

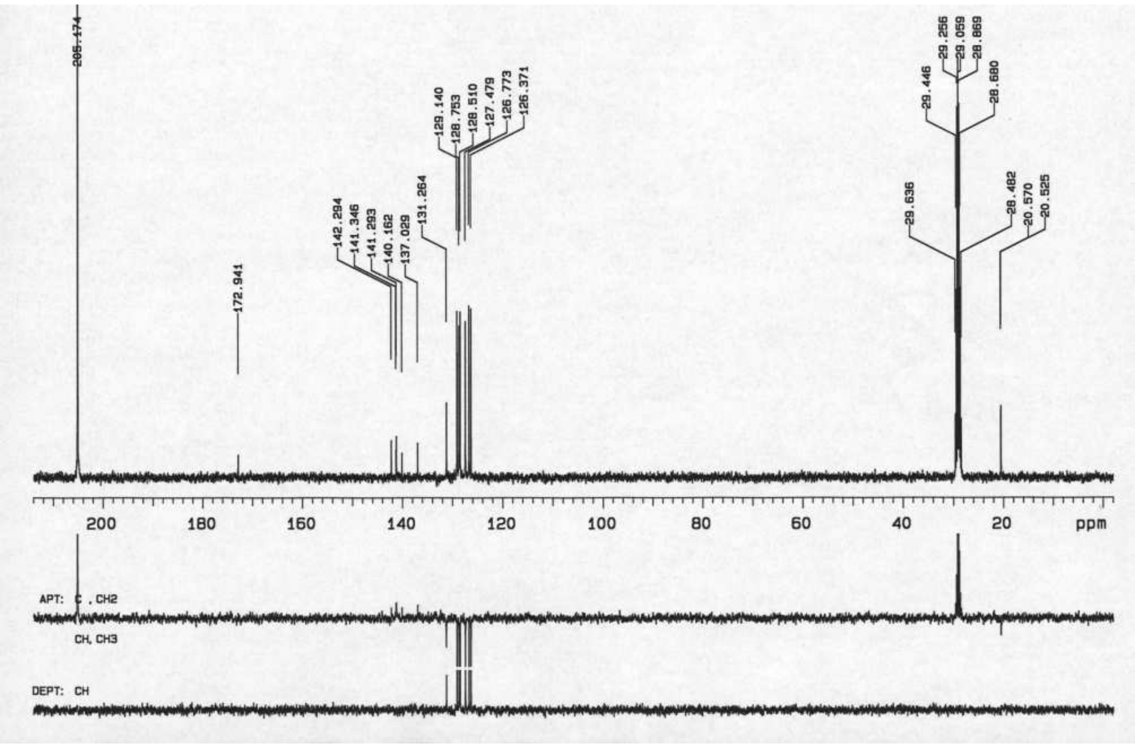

400 MHz, acetone-d_6

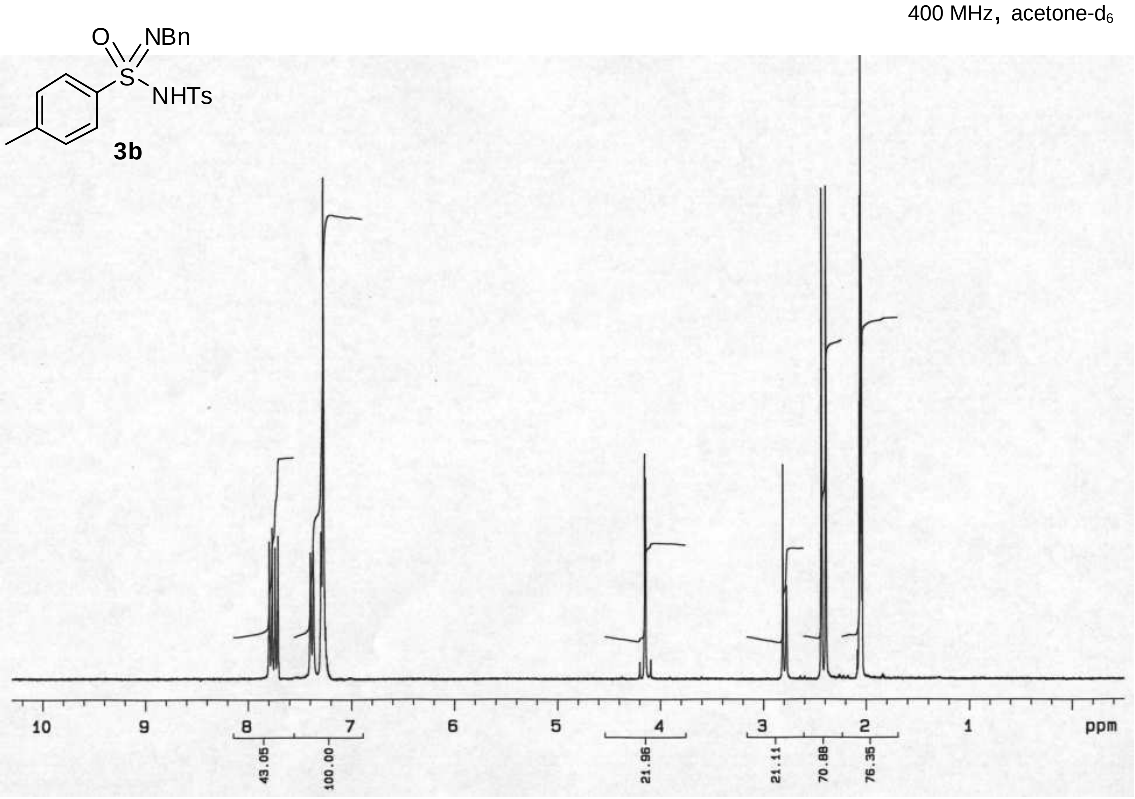

100 MHz, acetone-d_6

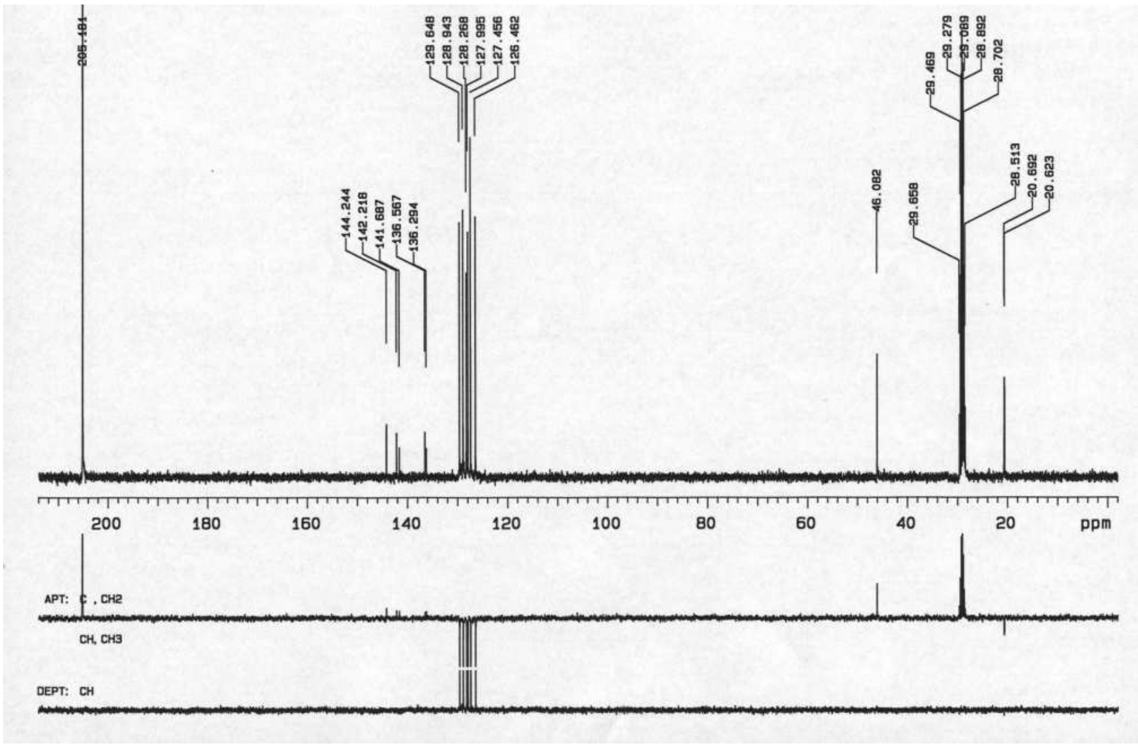

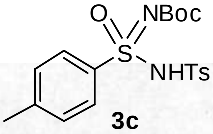

3c

300 MHz, CD_3OD

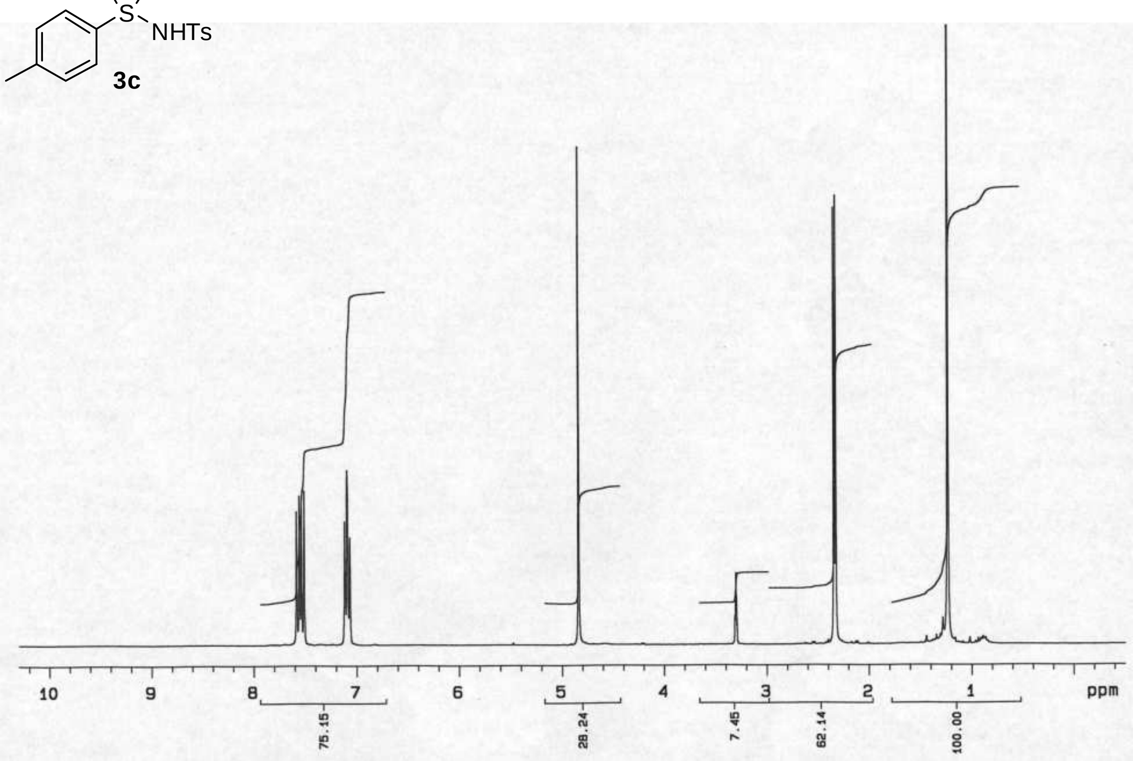

75 MHz, CD_3OD

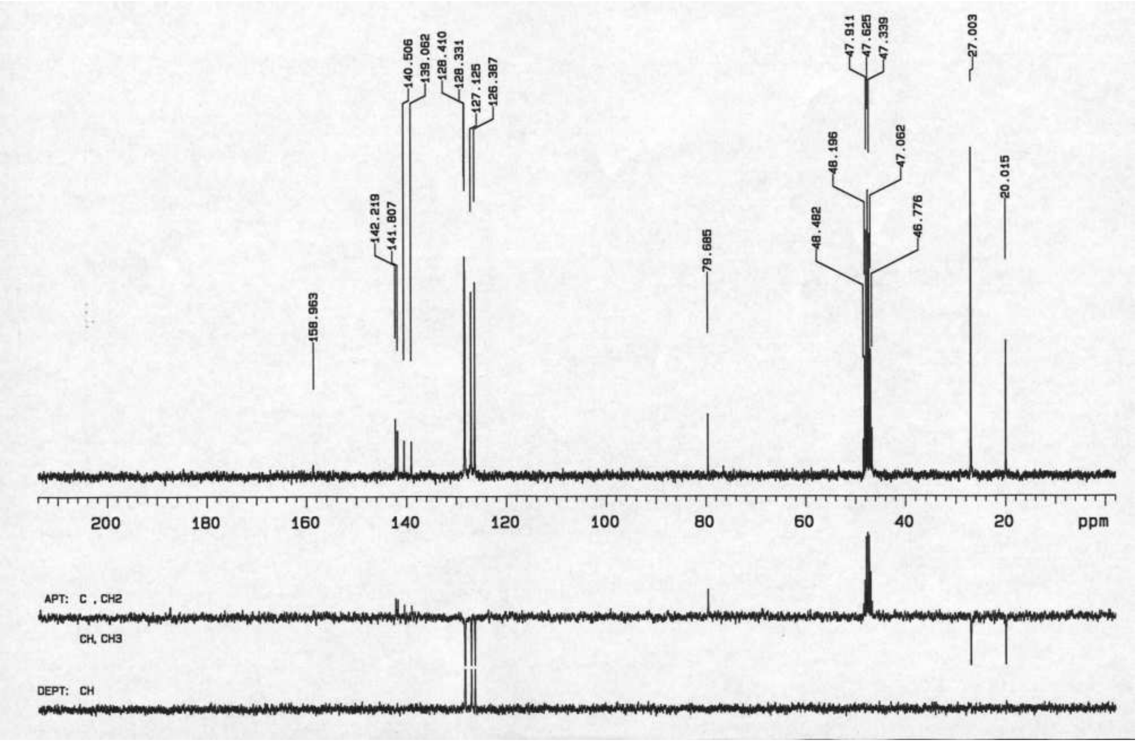

400 MHz，acetone-d_6

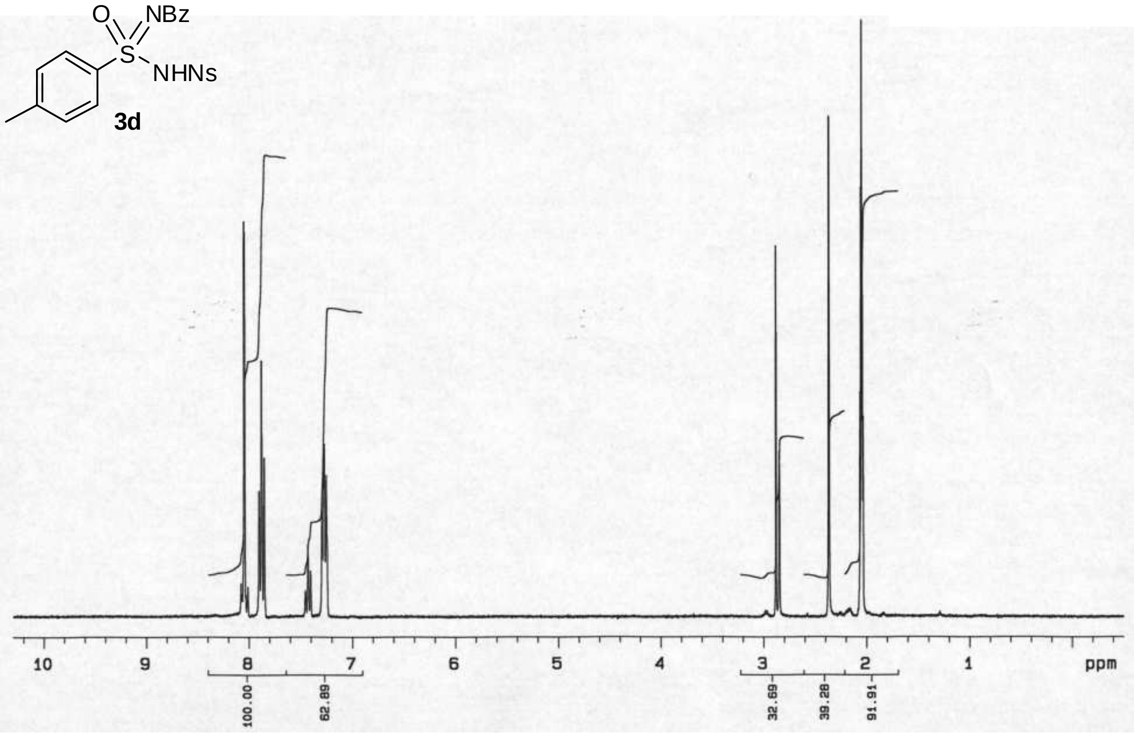

100 MHz，acetone-d_6

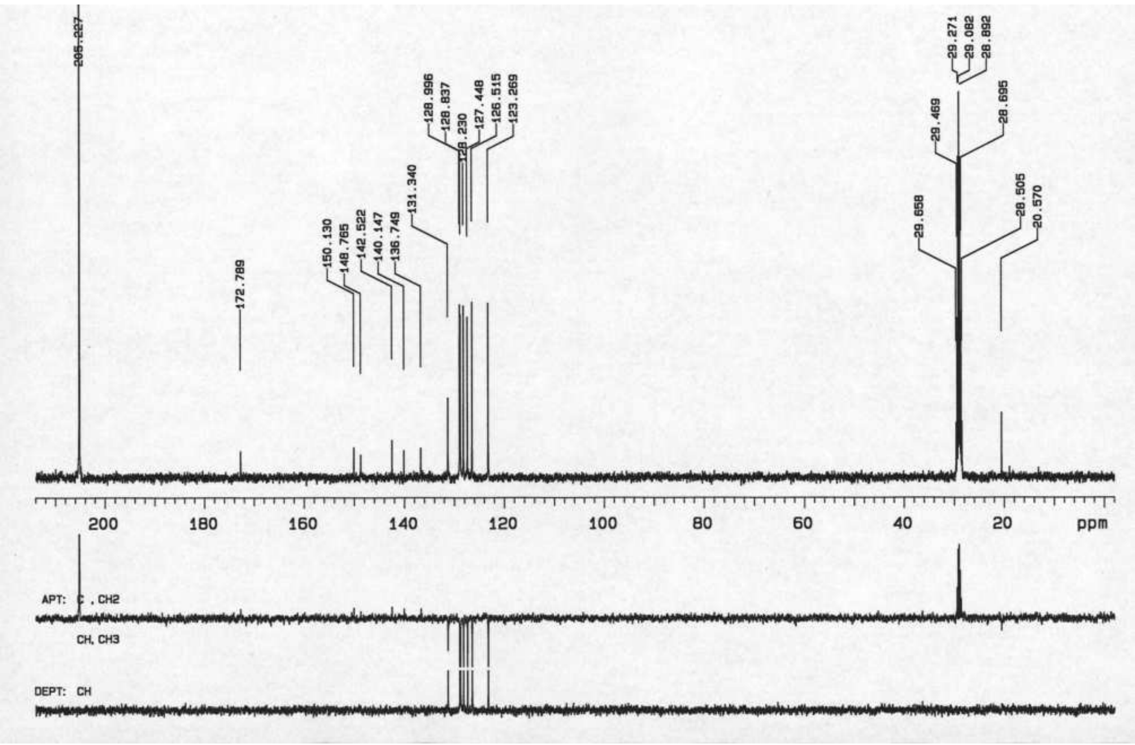

300 MHz, acetone-d_6

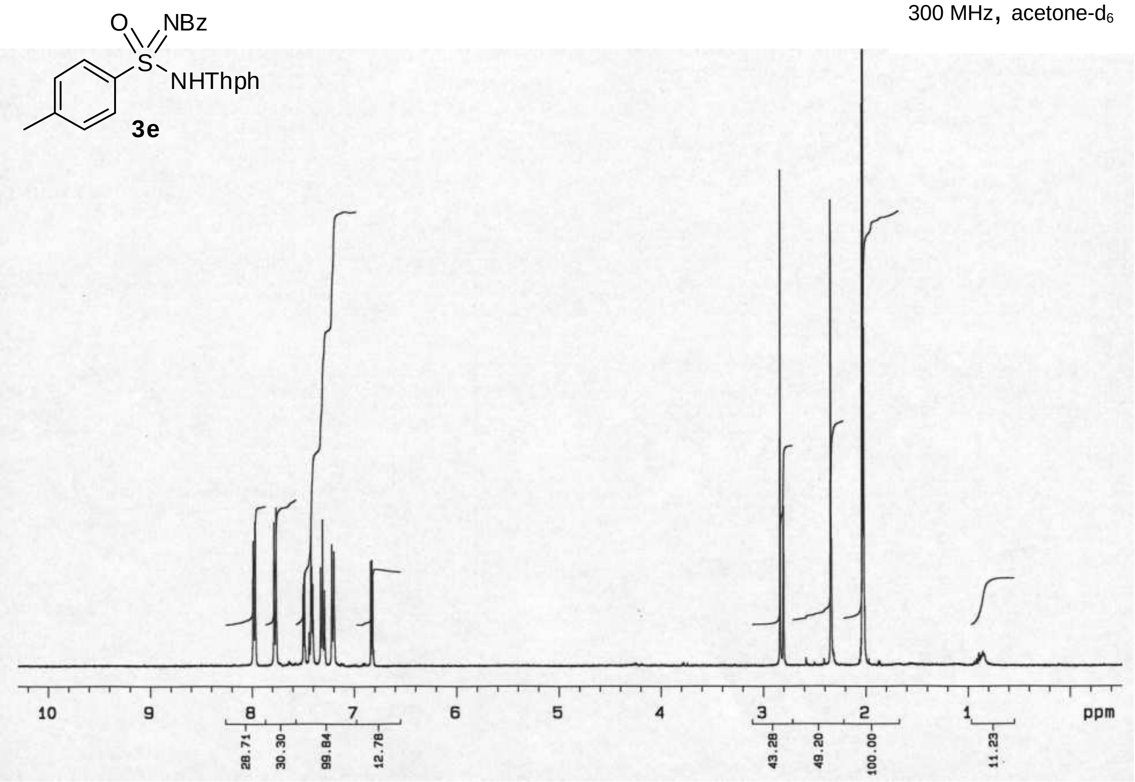

75 MHz, acetone-d_6

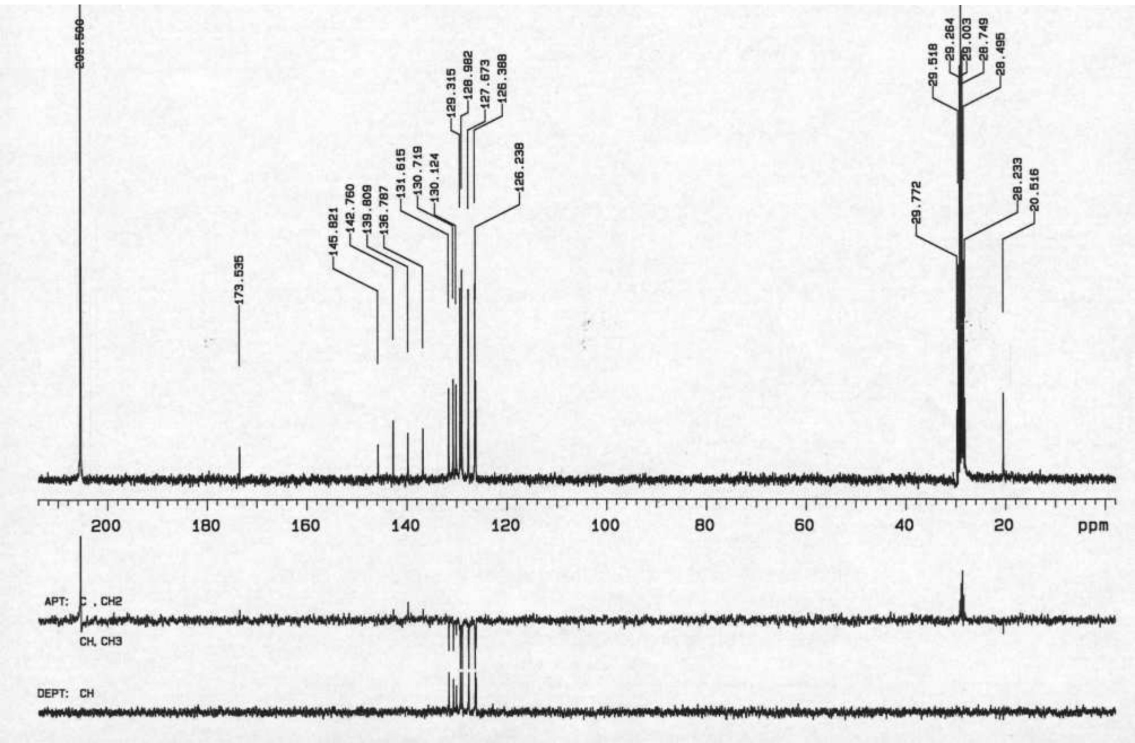

400 MHz, acetone-d_6

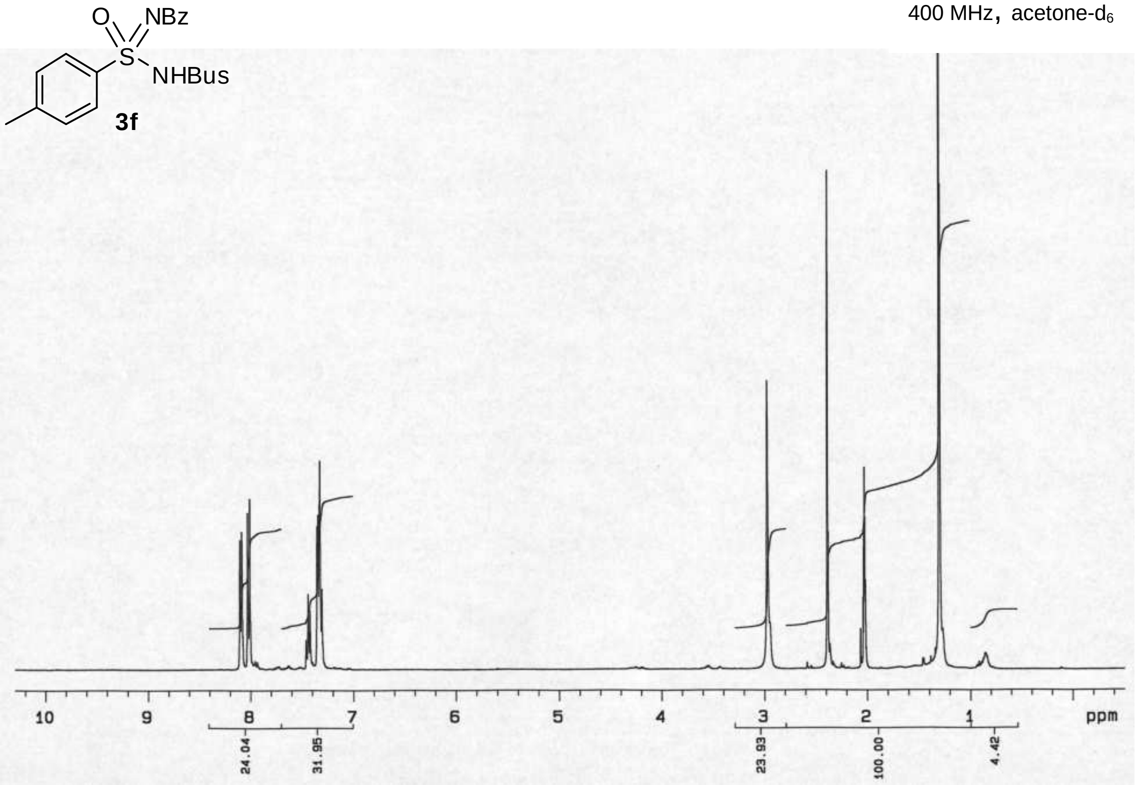

100 MHz, acetone-d_6

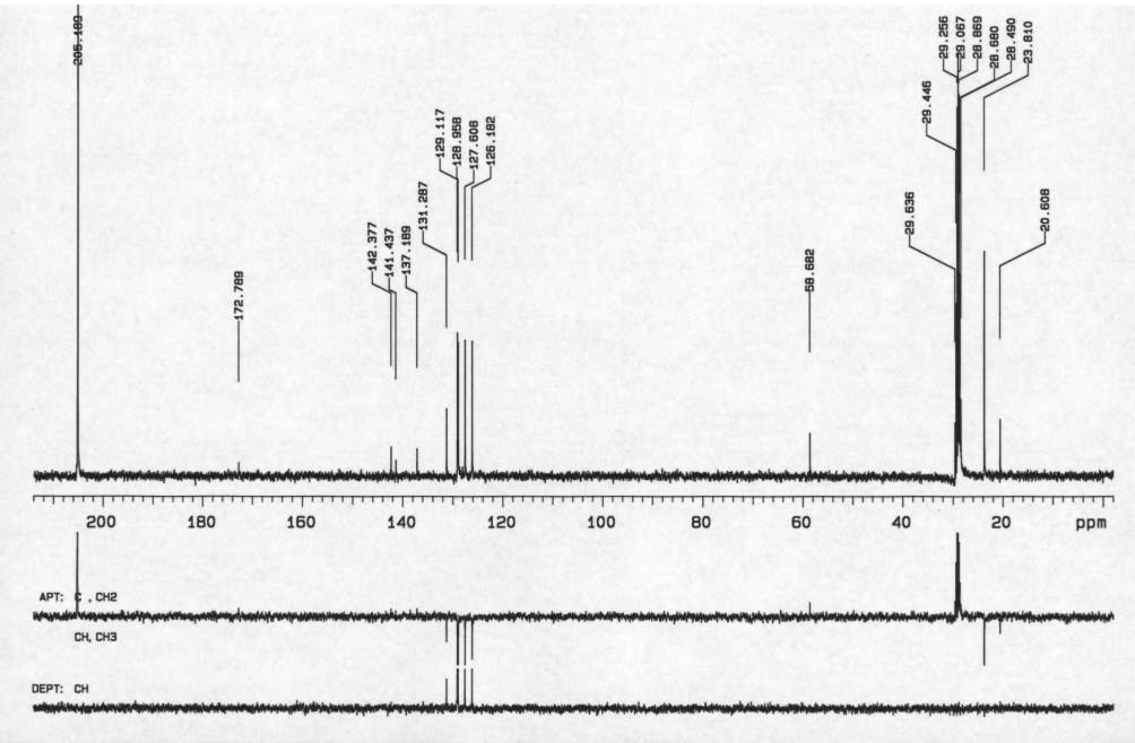

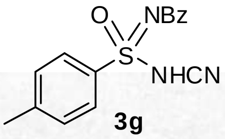

3g

300 MHz, acetone-d_6

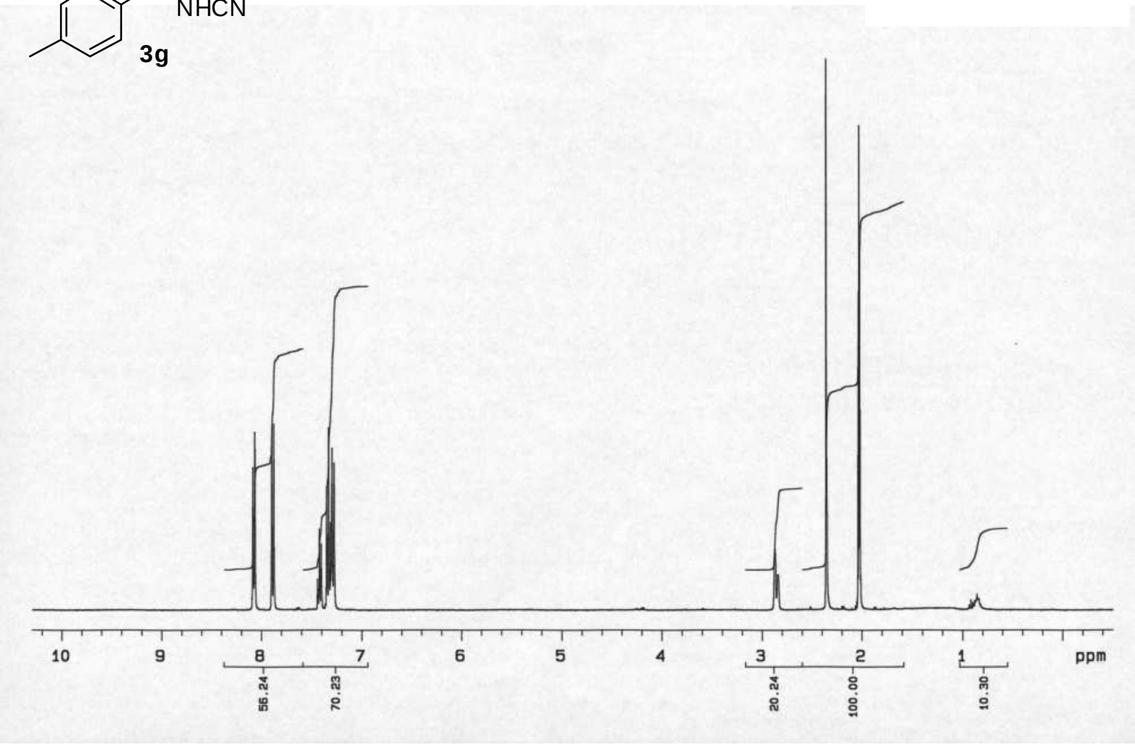

75 MHz, acetone-d_6

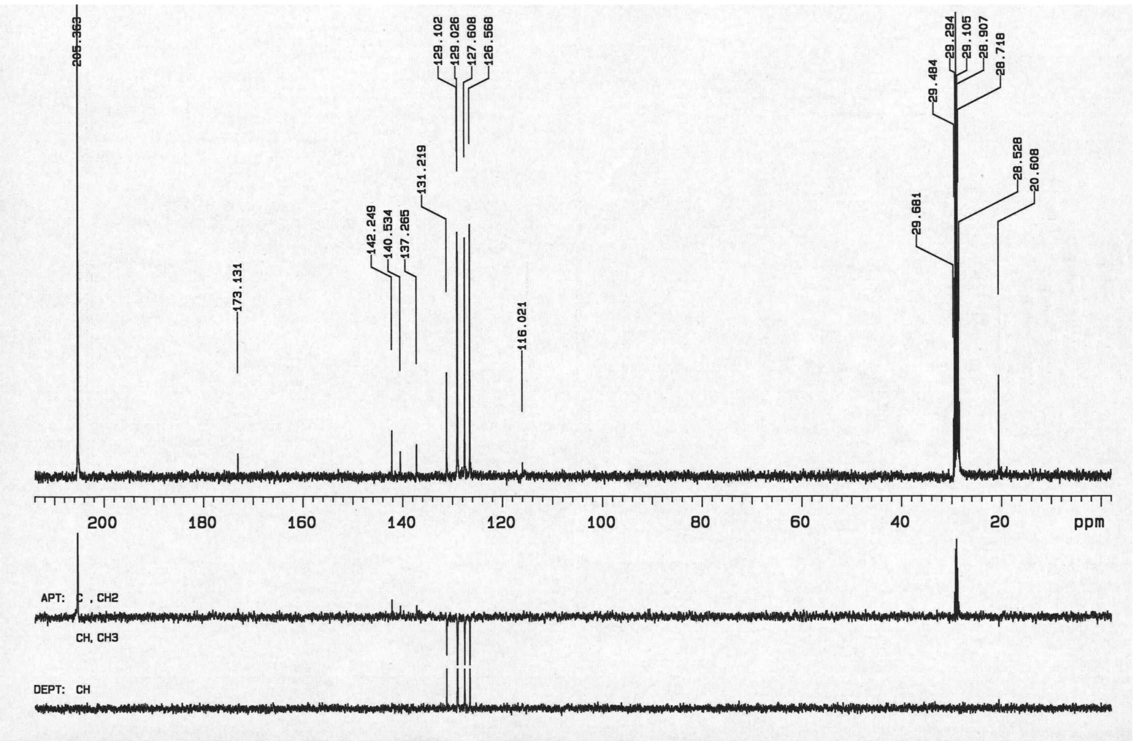

400 MHz, acetone-d_6

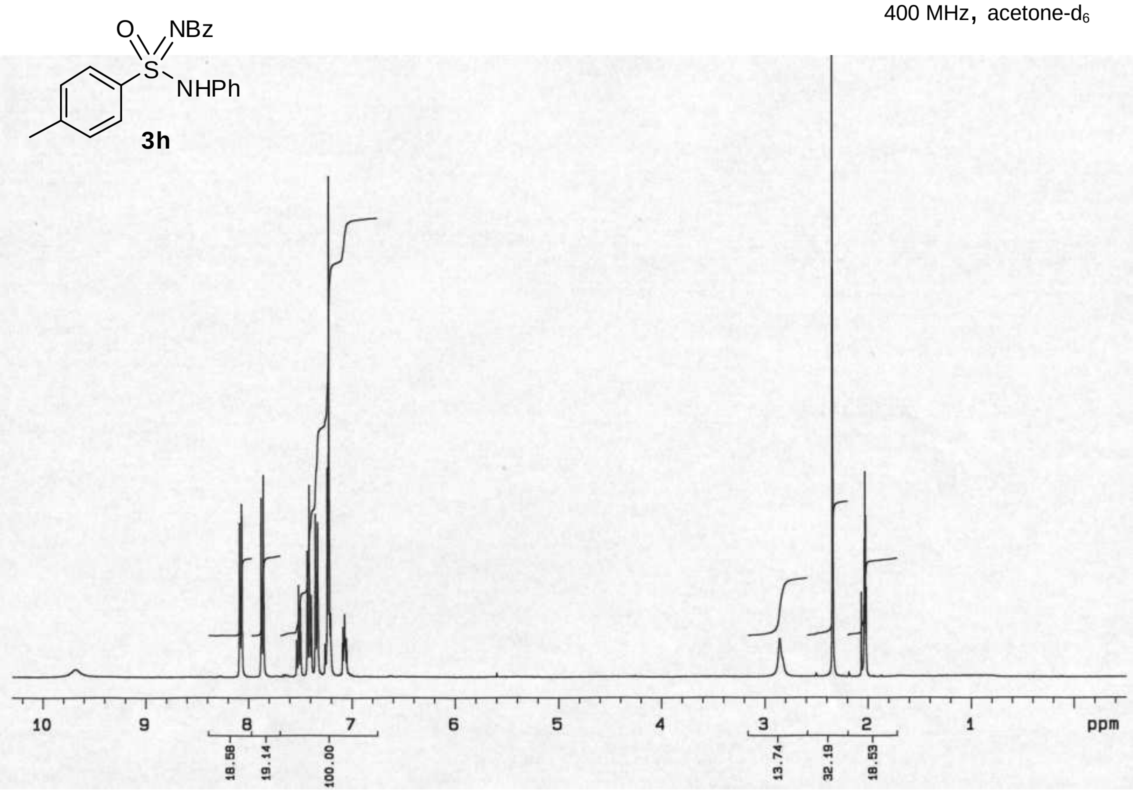

100 MHz, acetone-d_6

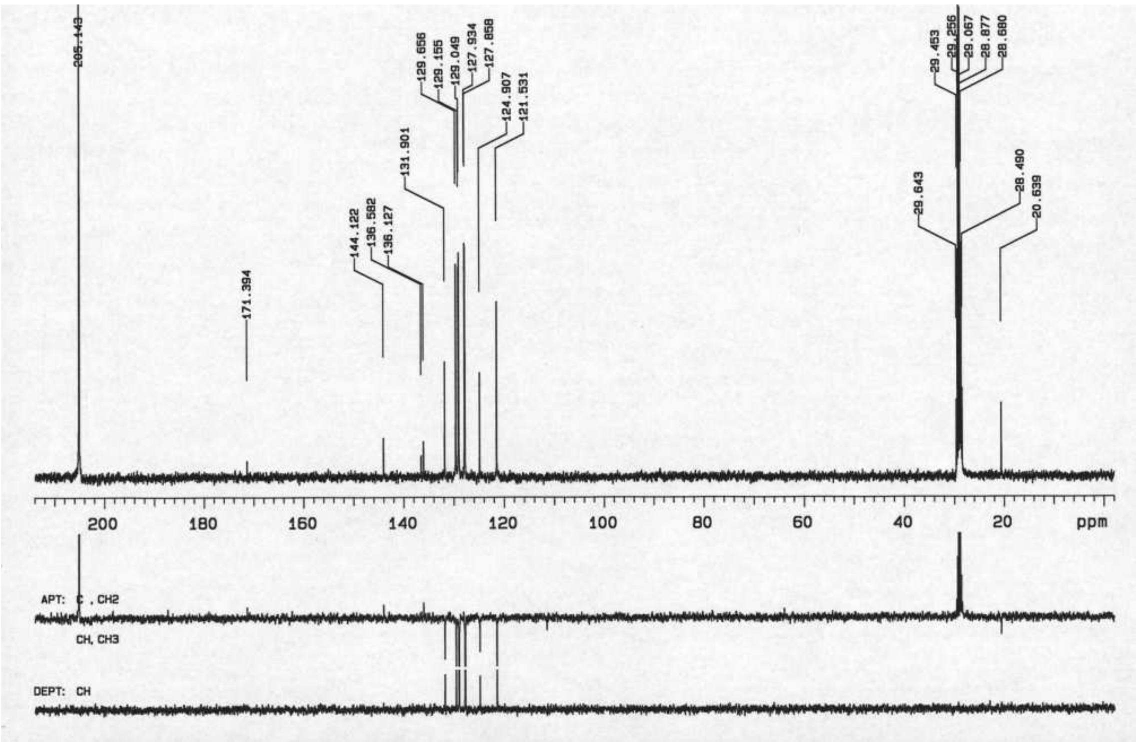

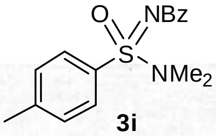

3i

300 MHz, acetone-d$_6$

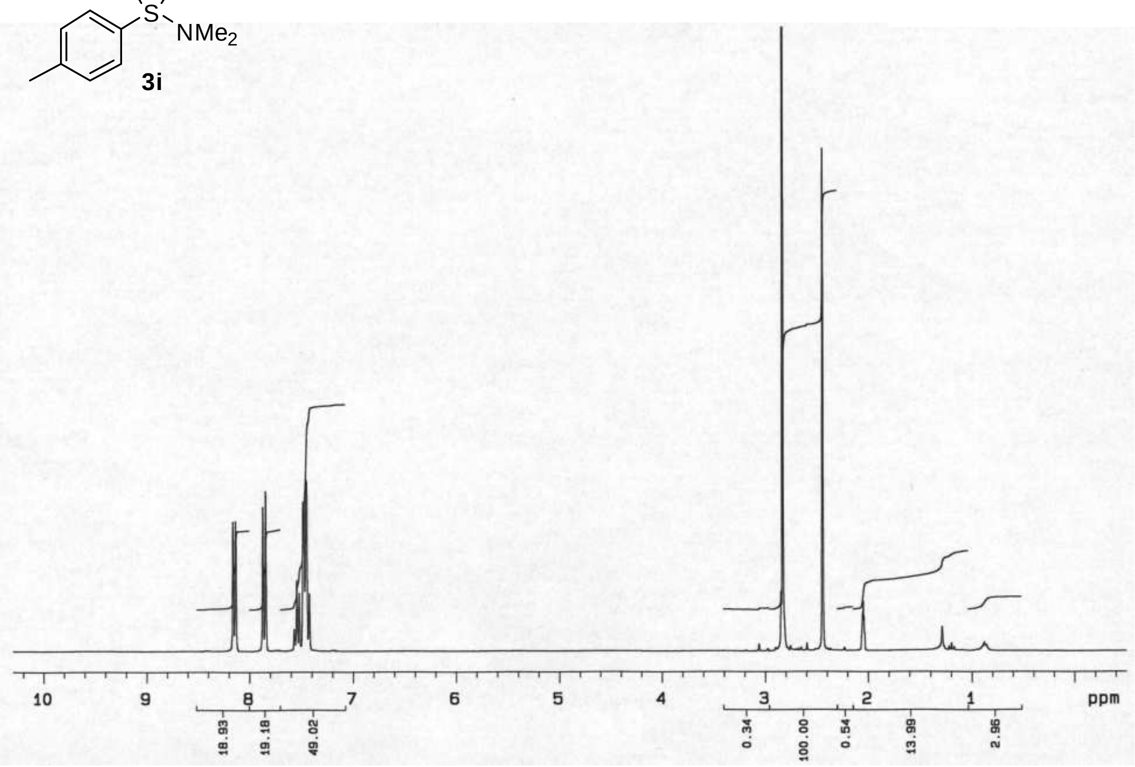

75 MHz, acetone-d$_6$

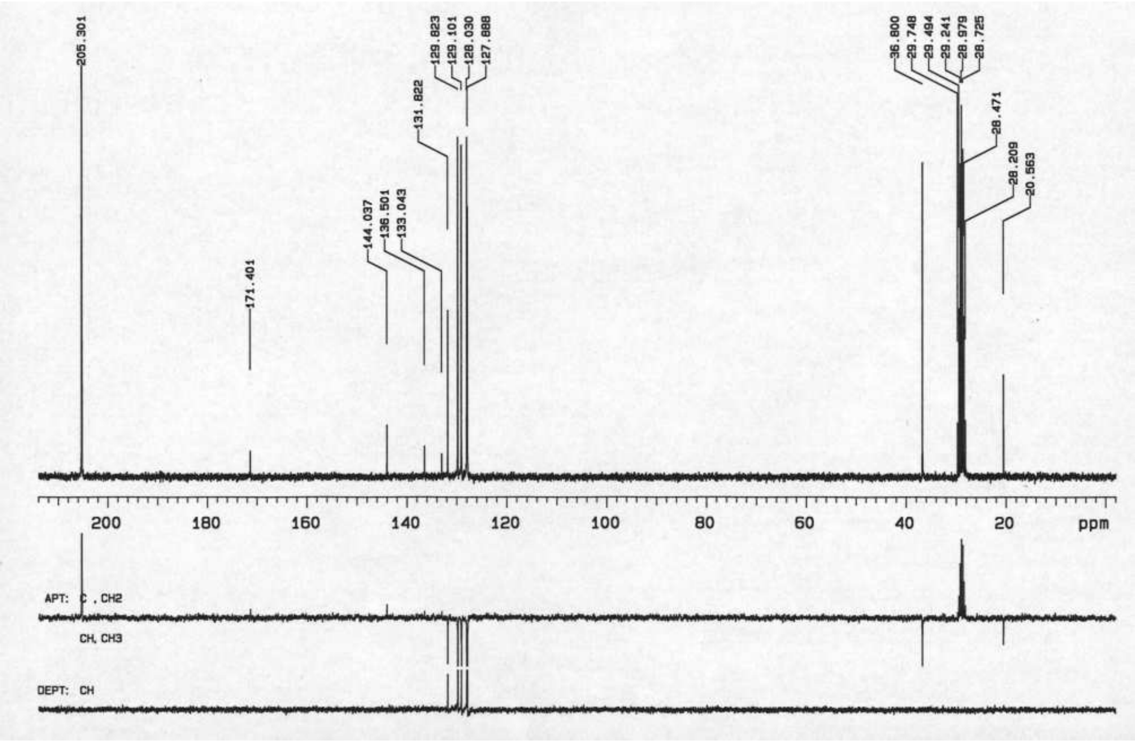

400 MHz, acetone-d_6

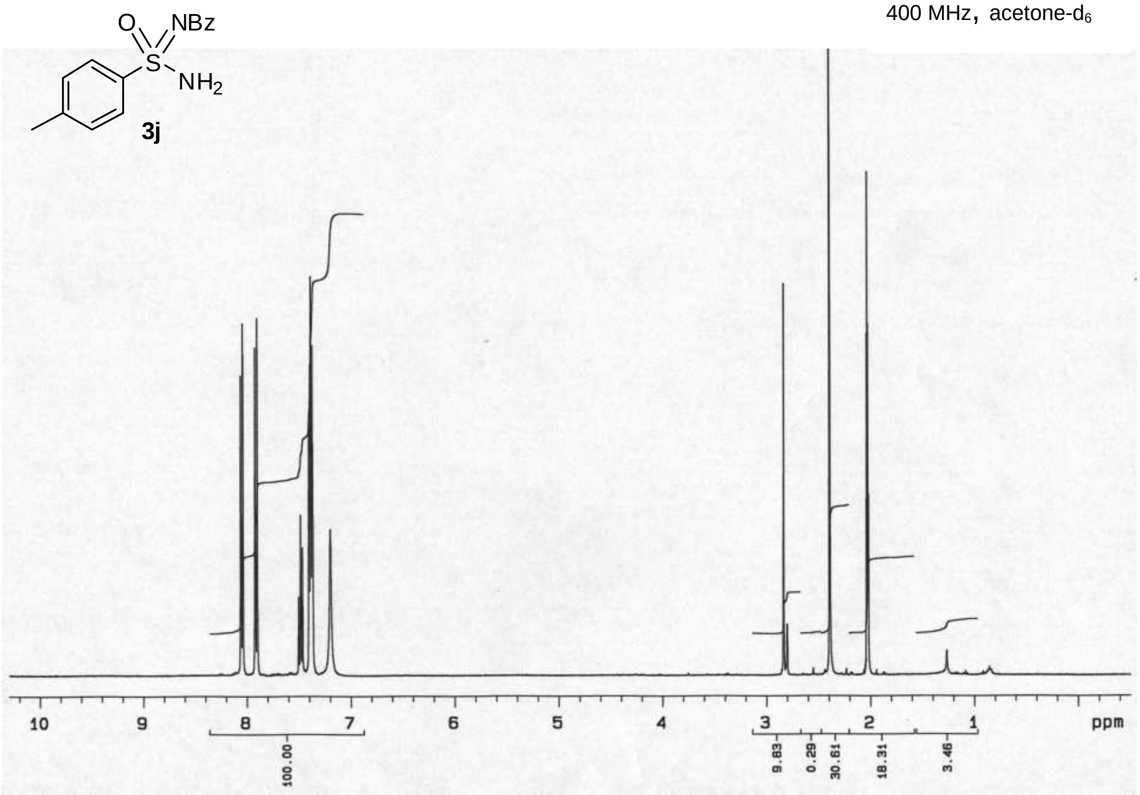

100 MHz, acetone-d_6

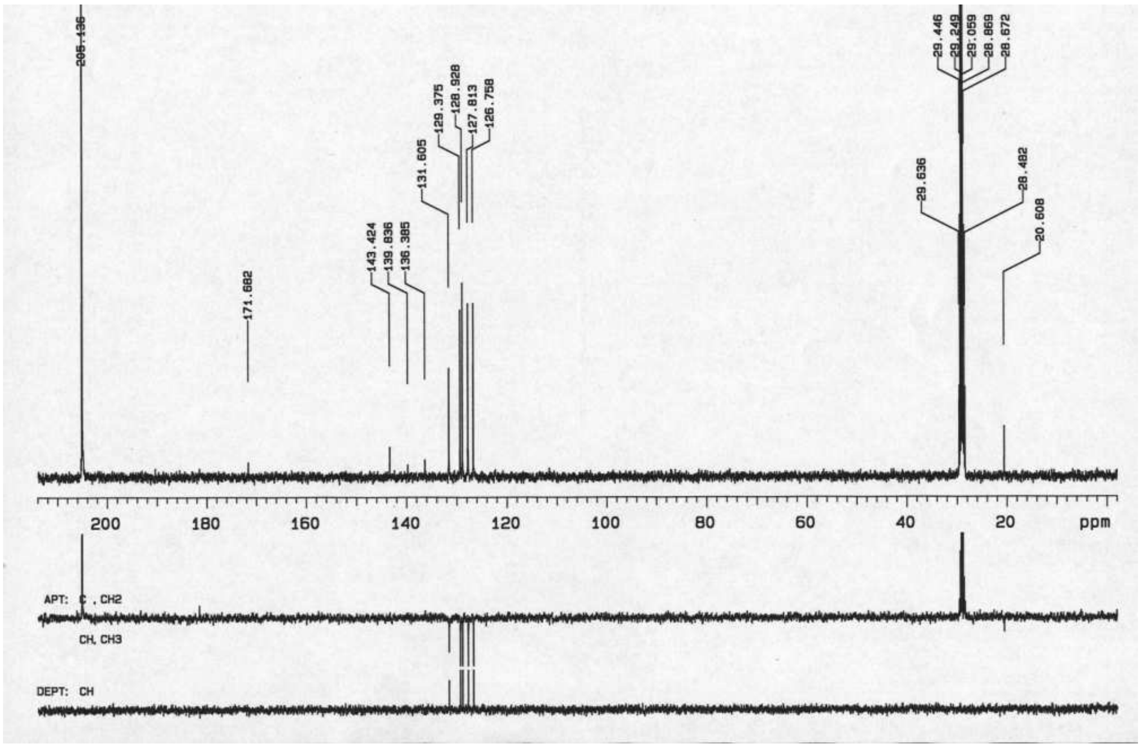

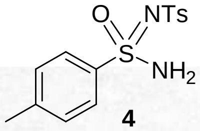

4

400 MHz, acetone-d6

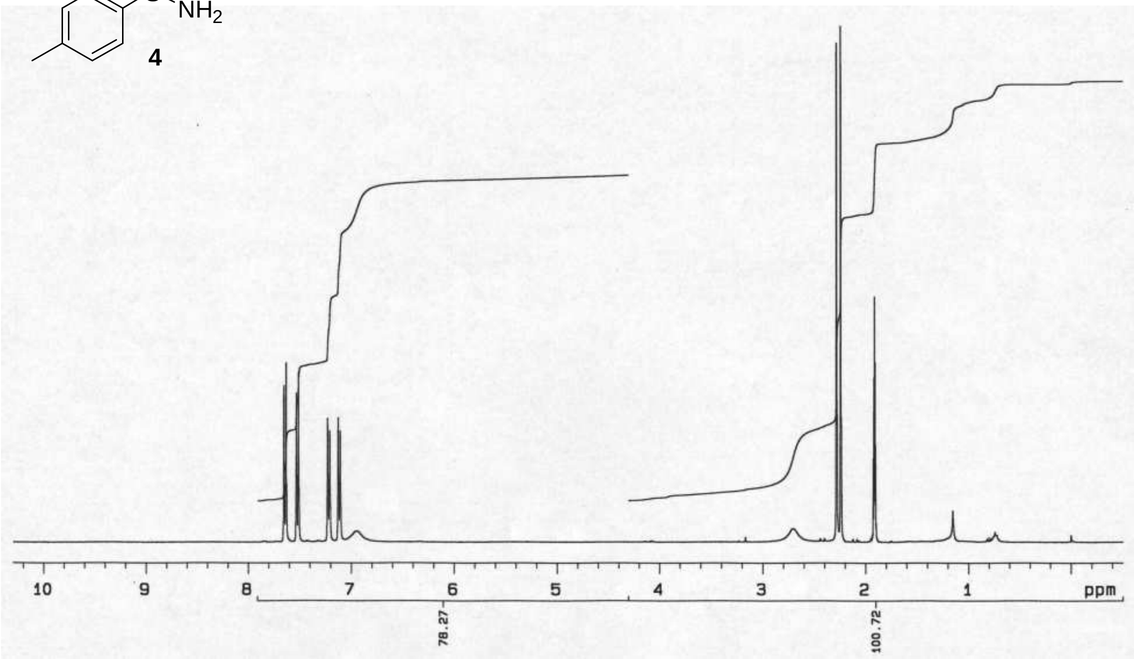

100 MHz, acetone-d6

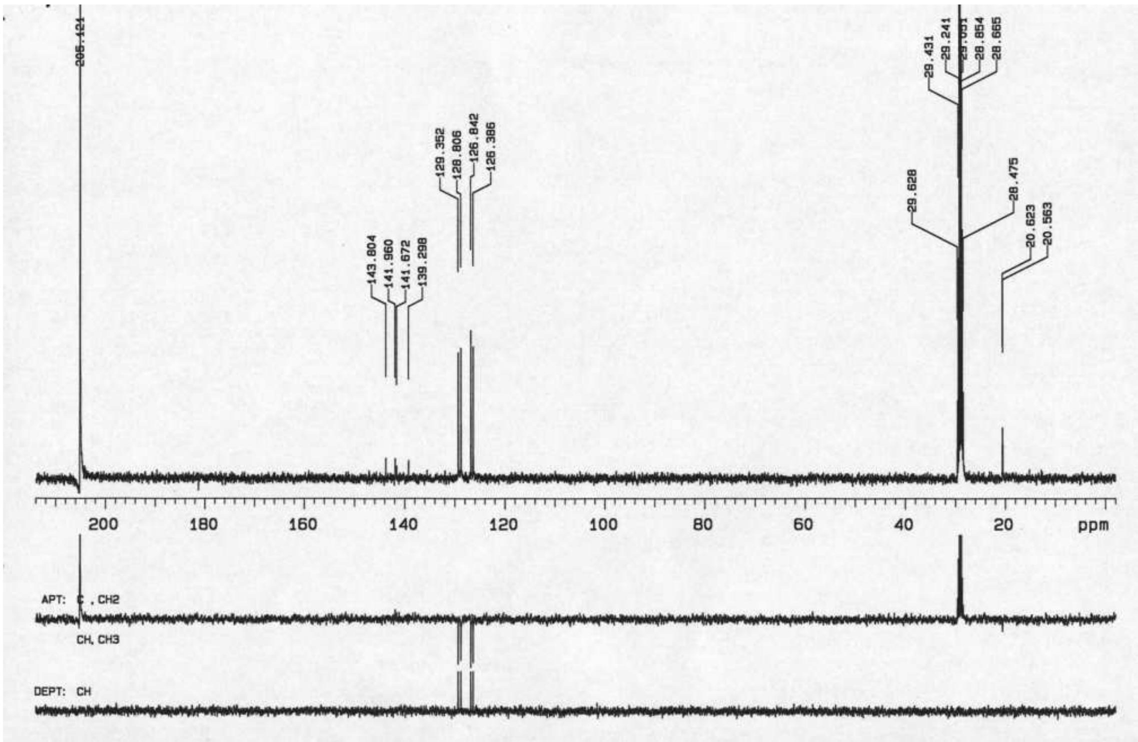

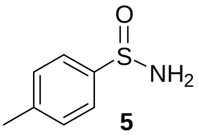

5

300 MHz, acetone-d_6

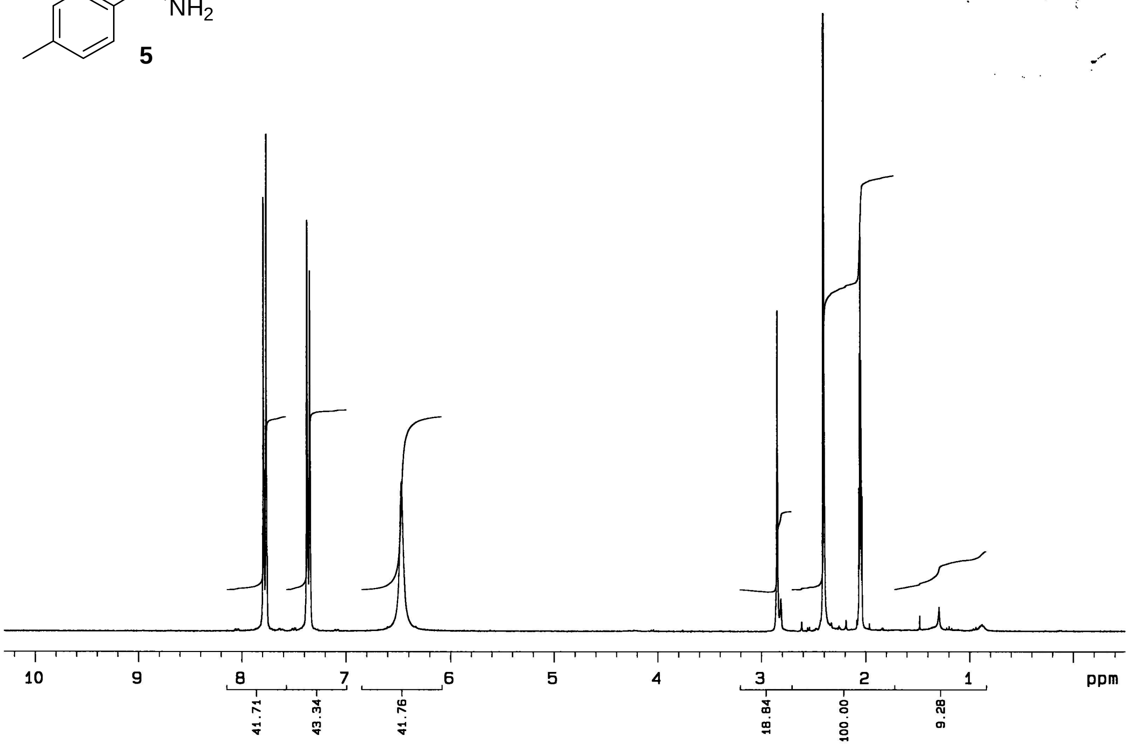

75 MHz, acetone-d_6

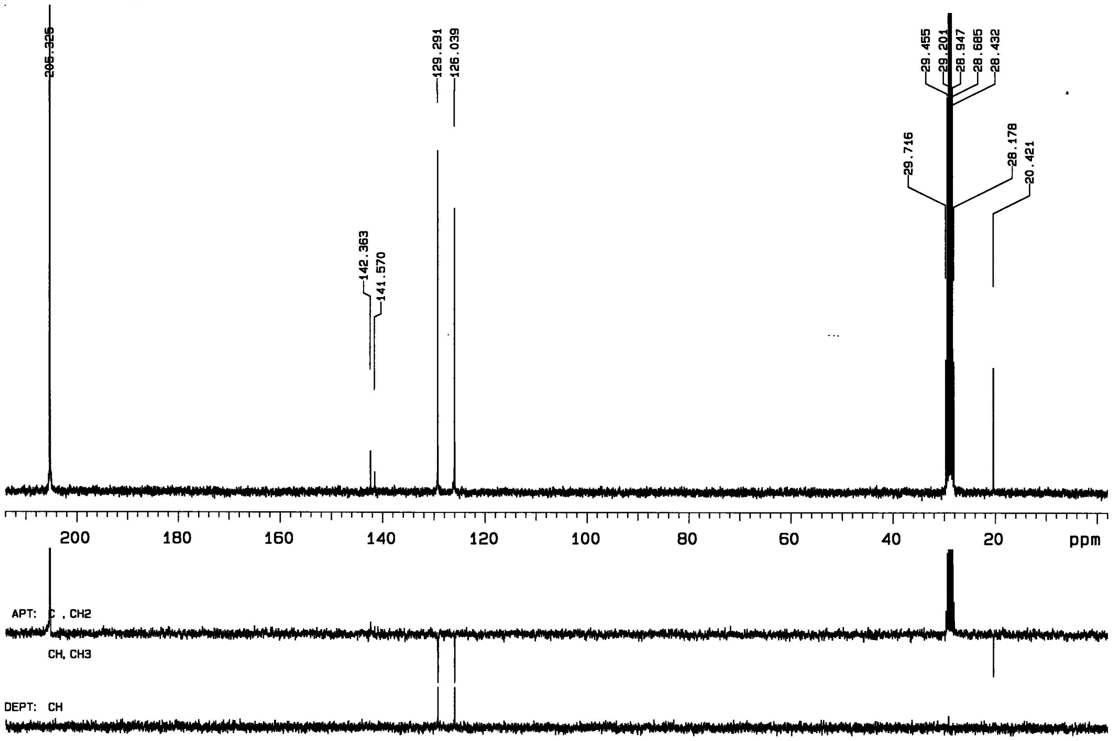

Supplement: File 1 — Synthesis of Sulfonimidamides from Sulfinamides by Oxidation with N-Chlorosuccinimide. Experimental Section. Experimental procedures, characterization of new compounds and 1H and 13C NMR spectra. [file Beilstein_J_Org_Chem-03-25-s001.pdf]
